# A Case of Impending Central Retinal Vein Occlusion Associated With Antiphospholipid Antibody Syndrome Complicated by Paracentral Acute Intermediate Layer Maculopathy

**DOI:** 10.7759/cureus.37256

**Published:** 2023-04-07

**Authors:** Yasuyuki Takai, Kenji Inoue

**Affiliations:** 1 Ophthalmology, Inouye Eye Hospital, Tokyo, JPN; 2 Opthalmology, Inouye Eye Hospital, Tokyo, JPN

**Keywords:** anti-cardiolipin antibodies, oct (optical coherence tomography), antiphospholipid syndrome, paracentral acute middle maculopathy, central retinal vein occlusion

## Abstract

We report a case of impending central retinal vein occlusion (CRVO) due to antiphospholipid syndrome (APS) complicated by paracentral acute middle maculopathy (PAMM). A 42-year-old man with no medical history acutely presented with blurred vision in his right eye. The best-corrected visual acuity was 20/20 in the right eye, but the Goldman visual field test showed a dark spot over the superior nasal region of the right eye. Fundus examination showed dilated and tortuous retinal veins of the right eye with delayed filling of the inferior retinal veins on fluorescein angiography. Optical coherence tomography (OCT) showed a hyperreflective lesion confined to the inner nuclear layer (INL), consistent with PAMM. After several weeks, his symptoms improved, and the dilated retinal vein and the INL hyperreflexia on OCT were reduced. Multiple positive findings for anti-cardiolipin antibodies were confirmed; therefore, he was diagnosed with APS and treated with aspirin. If the impending CRVO is associated with visual impairment, the complication of PAMM should be considered. In the presence of retinal vasculopathy without atherosclerotic factors, APS should be considered.

## Introduction

Paracentral acute middle maculopathy (PAMM) is a middle retinal layer imaging sign characterized by a hyper-reflective area of the inner nuclear layer (INL) on optical coherence tomography (OCT) [1]. Although the exact mechanism remains unclear, it has been hypothesized that ischemia of the deep retinal capillary plexus may be responsible.

Central retinal vein occlusion (CRVO) is a cause of sudden visual loss and is classified as either ischemic or non-ischemic depending on the presence of retinal ischemia. In some cases, macular edema may develop, thereby leading to visual dysfunction. Impending CRVO exhibits no hemorrhage or only a few hemorrhages with dilated and tortuous vessels and is rarely associated with visual disturbance [2]. Risk factors for CRVO include hypertension, diabetes mellitus, aging, and other atherosclerosis-related conditions [3].

Antiphospholipid syndrome (APS) is an autoimmune thrombosis caused by antiphospholipid antibodies and often leads to various thrombotic events such as cerebral infarction, deep vein thrombosis, or complications during pregnancy. Several retinal circulatory disorders, such as CRVO or ischemic optic neuropathy, have been reported in association with APS [4].

The present report describes a case of APS presenting with an impending CRVO with PAMM.

## Case presentation

**Figure 1:**
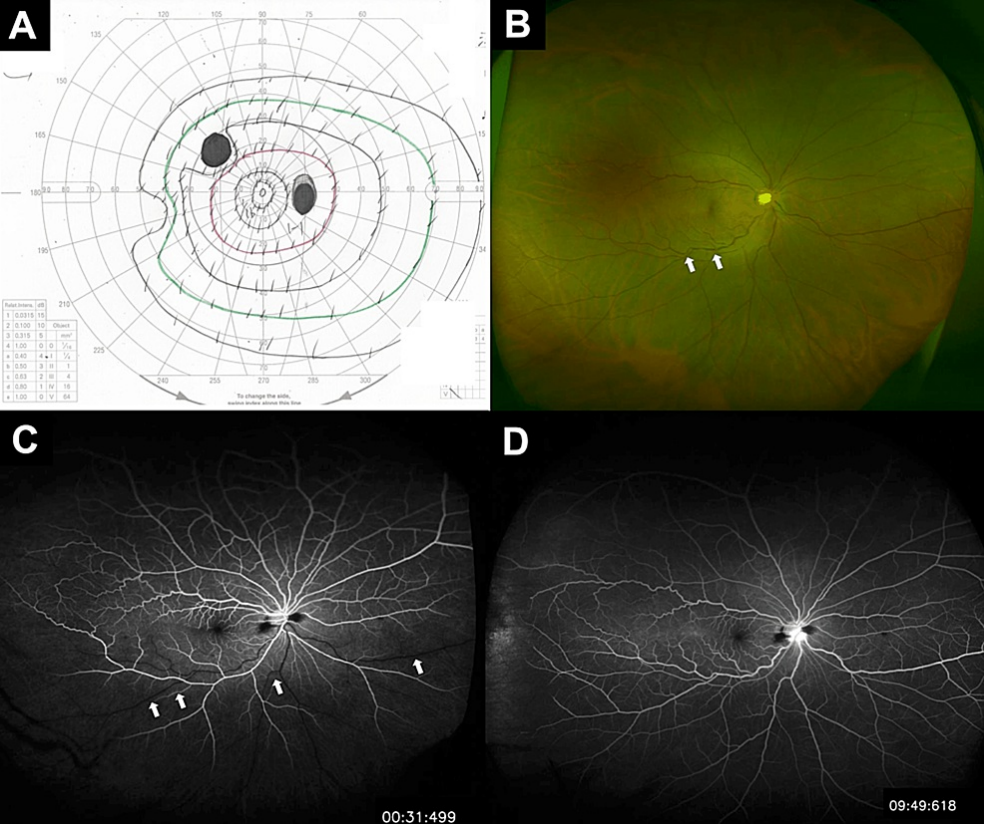
Findings at the initial examination The Goldmann visual field test demonstrated that there was a dark spot over the nasal side of the right eye (A). The fundus examination showed the presence of significant inferior tortuous and dilated retinal veins (B, white arrows). Fluorescein angiography indicated there were dilated and tortuous inferior retinal veins during the early phase, with delayed inflow (C, white arrows), and no presence of non-perfusion areas during the late phase (D).

A 41-year-old man with no relevant medical history noticed sudden blurred vision in the upper field of his right eye. As his symptoms did not improve, he visited our clinic on the third day since the onset. At the initial examination, the best-corrected visual acuity (BCVA) was 20/20 OU, and the intraocular pressure was 14 mmHg OU. A Goldman visual field test revealed a dark spot over the superior nasal region of the right eye (Figure 1A). Slit-lamp examination was normal. Funduscopic examination revealed dilated and tortuous veins in the right eye (Figure 1B), and fluorescein angiography revealed delayed inflow in the inferior veins as compared to the superior veins (Figure 1C). However, there was no non-perfusion area during the late phase (Figure 1D).

**Figure 2:**
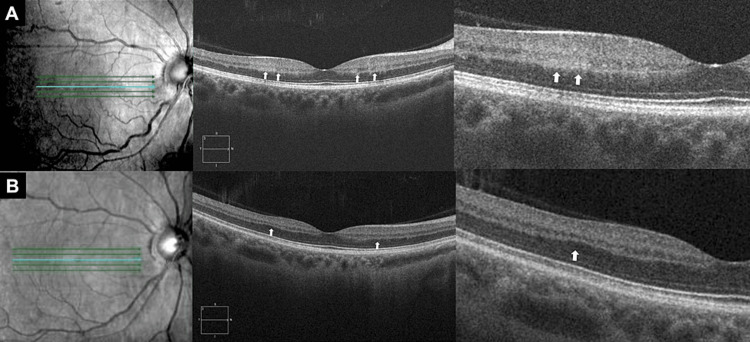
Optical coherence tomography findings Optical coherence tomography demonstrated there was a hyperreflective lesion that was limited to the inner granular layer (A, white arrows). After four months, the retinal ischemic perivascular lesions were shown to be mildly atrophic (B, white arrows).

OCT revealed the presence of a hyper-reflective lesion that was confined to the INL in the parafoveal area of the right eye (Figure 2A), leading to the diagnosis of PAMM due to an impending CRVO. The patient's symptoms improved over the course of several weeks, and his upper visual field defect disappeared. A few days later, a fundus examination showed there was a slight hemorrhage around the optic disc in the right eye, which spontaneously resolved, followed by a reduction in the tortuous, dilated venous findings. OCT examination demonstrated that the hyper-reflective findings in the INL had disappeared and the retinal ischemic perivascular lesions were mildly atrophic after four months from onset (Figure 2B).

Blood tests revealed elevated anti-cardiolipin antibodies at a level of 13.1 U/mL. As positive findings for anti-cardiolipin antibodies were recognized on three consecutive occasions at the Department of Internal Medicine, he was subsequently diagnosed with APS. To prevent a recurrence, the patient was treated with aspirin and has been free of thrombotic events to date.

## Discussion

We observed a case of PAMM due to impending CRVO in an adult male without atherosclerotic factors. After the spontaneous resolution of the PAMM, his symptoms improved. After searching for the cause of the thrombosis, a diagnosis of APS was made. As impending CRVO is unlikely to cause vision loss [2], PAMM should be considered a complication when patients present with visual impairment. APS should also be considered during the differential diagnosis of retinal vasculopathy in patients without atherosclerotic factors.

The retinal capillary network is a multilayered capillary network consisting of radial peripapillary capillaries (RPC), superficial capillary plexus (SCP), and deep capillary plexus (DCP) [5]. PAMM is associated with local circulatory failure of the DCP, with capillary loss in the DCP previously reported in an OCT angiography study [6]. The acute phase of PAMM is associated with hyper-refractive lesions of the INL, whereas the chronic phase of PAMM results in a thinning of the INL, which suggests that there is a local circulatory defect in the INL [7]. Acute macular neuroretinopathy (AMN), which causes hyperintense lesions in the outer nuclear layers and the outer plexiform layers, and branch retinal artery occlusion (BRAO), can be identified via a differential diagnosis. OCT is useful for this differentiation, as BRAO is distinguished by the fact that RPC, SCP, and DCP all appear as full-thickness edema, with the respective retinal layers becoming thinner in the chronic phase [8]. PAMM is often undetectable with fluorescence angiography and should be evaluated with OCT.

In addition to CRVO, PAMM may be caused by localized retinal ischemia, which includes central retinal artery occlusion (CRAO), BRAO, diabetic retinopathy, hypertensive retinopathy, and Purtscher’s retinopathy [7,9]. Systemic diseases such as AL amyloidosis, migraine, medications (amphetamines, caffeine, vasodilators, oral contraceptives), orbital compression trauma, decreased blood flow, and post-vaccination-associated changes have also been reported to cause PAMM [7,10]. It has also been reported recently that there were cases of PAMM following a COVID-19 infection [11]. Because of the variety of underlying diseases, it is important to search for definitive causes when PAMM is diagnosed.

APS can lead to a variety of ocular complications [4]. The most common of which are retinal circulatory disturbances, including CRAO and CRVO. Corneal ulceration, scleritis, and uveitis have also been reported. In addition to ocular complications, a variety of systemic vascular occlusions can potentially occur, including cerebral infarction or deep vein thrombosis [12]. APS can cause thrombosis of both arterial and venous vessels, and cases of concurrent CRAO and CRVO have been reported [13]. The diagnosis is based on the presence of thrombosis and should include blood tests for anticardiolipin antibodies and anti-β2-glycoprotein antibodies, with lupus anticoagulants included, and the tests should be positive at least twice [14]. If the patients develop thrombosis in the absence of atherosclerotic factors, are young, or have recurrent retinal vasculopathy, then APS should be considered during the differential diagnosis. As thrombosis may occur outside the eye, patients suspected of APS must be closely monitored and treated in collaboration with physicians to prevent a recurrence.

## Conclusions

PAMM is a feature of ischemic retinal disease such as CRVO and should not be overlooked in the setting of visual impairment. APS should be considered during the search for the cause of retinal vasculopathy without atherosclerotic factors.
